# Innovation in e-learning: learning for all

**DOI:** 10.3332/ecancer.2014.467

**Published:** 2014-09-30

**Authors:** K Butcher, R Bamford, D Burke

**Affiliations:** 1 Severn Postgraduate School of Surgery, Health Education South West, Severn Postgraduate Medical Education Deanery House, Unit D-Vantage Business Park, Old Gloucester Road, Bristol, BS16 1GW, UK; 2 *e*cancer, 154 Cheltenham Road, Bristol, BS6 5RL, UK

**Keywords:** online education, medical education, pancreatic cancer, collaboration, surgical oncology, ePOSSOM

## Abstract

Online learning is not a new concept for most in the medical profession. However, surgical oncology is poorly represented, and in a world of ever-changing research evidence, relying on published texts may not be efficient learning or an accurate representation of current practice for many trainees. This article demonstrates how our educational collaborative, ePOSSOM, approaches the problem. It outlines the development process of the whole project between *e*cancer and the Severn School of Surgery, UK, and provides links to the pilot completed modules on pancreatic cancer and its treatment for the reader to experience.

## Background and purpose

Medical education is evolving, and the days of teaching being didactic are gone. Modern technology enables the learner to access educational material whilst mobile, at any time, and at any location. Distance learning has developed beyond text books and essay reports as a prime method of assessment [1]. The workload on trainees and the impact of the European Working Time Directive means that up-to-date online educational material can be invaluable, and its use has been supported by the Department of Health and the General Medical Council (GMC) [2, 3]. This indicates an exciting time for both learners and educators.

Surgical oncology is a fast-developing science, with advances in treatment becoming available almost weekly. Keeping up to date with this bank of best evidence can be time consuming and disjointed, leaving the learner no choice but to visit multiple resources. The availability of multimedia resources that combine the acquisition of evidence-based knowledge with educationally validated teaching methods and assessments are sparse, especially within this field.

The *e*POSSOM (*e*cancer POst graduate school of Surgery Surgical Oncology Modules) project was conceived to address this in an innovative e-learning approach. The following article describes our approach and our aims.

## Methods

A collaborative was set up between ecancer and the Severn Postgraduate School of Surgery, based in Bristol, UK, with support from the University of Bristol Medical Education department where needed. ecancer, the leading oncology channel, is an online open access resource for oncology research and information (www.ecancer.org). The Severn School of Surgery is part of the Health Education South West, the new organisation that has overarching responsibility for the planning, development, education, and training of the healthcare and public health workforce in the South West region of the UK. (http://southwest.hee.nhs.uk/).

The project began in 2013 with a core team whose members are identified in Table 1. This group developed the aims of the collaborative to create high-quality, cost-effective, evidence based, and educationally rigorous e-learning modules for organ-specific cancer. The content was aimed at the level expected of surgical trainees taking their final exams, but it will be accessible to any interested party. Importantly, it was decided that the project should be trainee led, and hence this resulted in Miss Butcher taking the role of Pilot Project Lead.

Recruitment of interested collaborators began by advertising to specialist and core surgical trainees within the training region to create a team of ten trainees. The project was designed for trainees to work in pairs of a senior and a core trainee, with the senior trainee acting as a mentor. The purpose of this was to enhance the critical appraisal of the evidence and educational theory. Each pair was assigned a module to develop, which would be linked together to create the final project.

**Figure 1.**
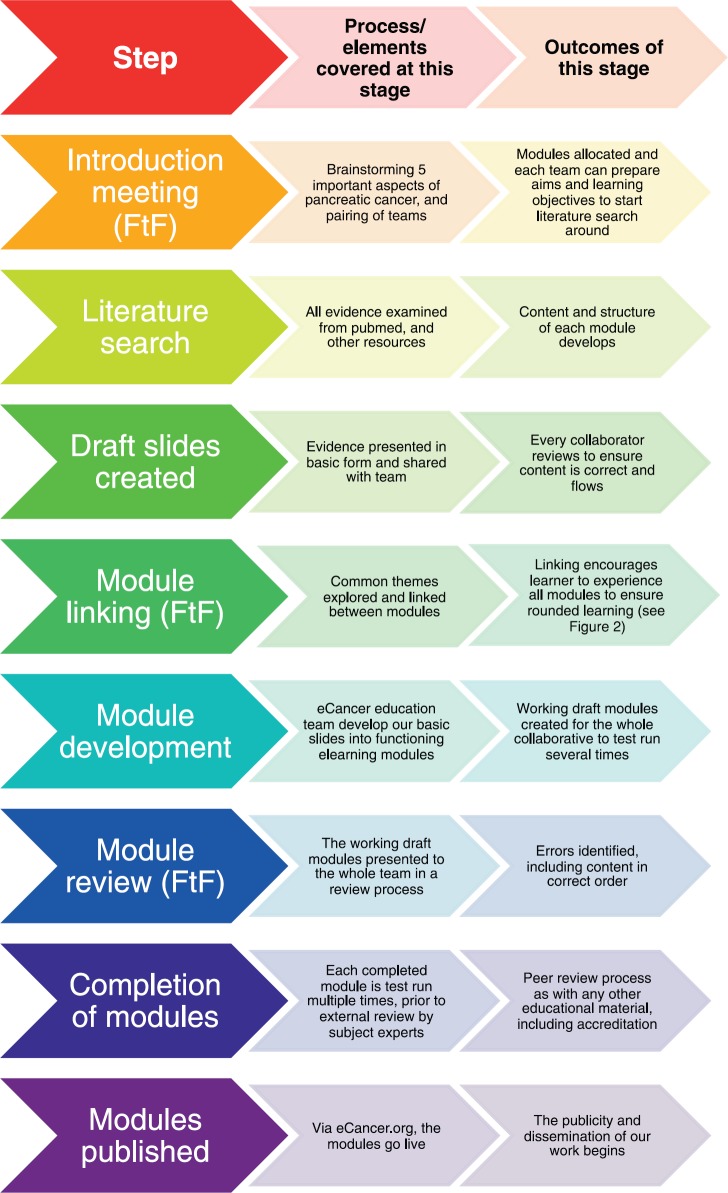
The process of developing the pilot *e*POSSOM project. FtF: face-to-face meeting.

The development process required the use of face-to-face meetings and individual work to create the content for each module and to develop the educational style with which they wished it to be presented. The *e*cancer educational team developed the technical aspect of the modules. This process is highlighted in Figure 1.

## Results

Eighteen months after the collaboration began, we are delighted to be able to present our completed modules: http://ecancer.org/education/course/7-pancreatic-cancer-surgery-course-for-post-graduate-surgical-trainees.php.

**Figure 2.**
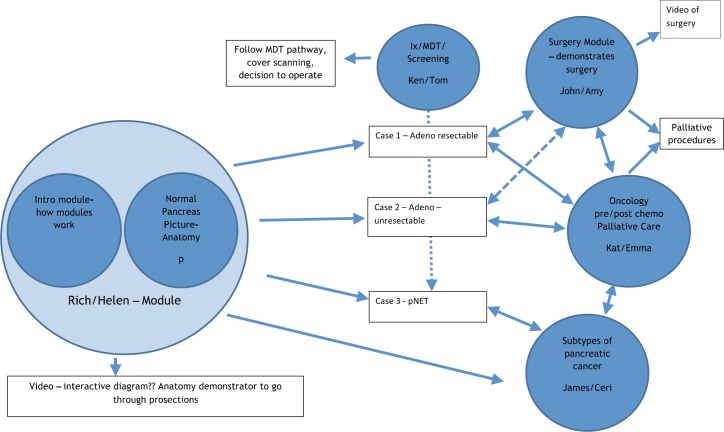
An example of how the components are linked.

**Figure 3.**
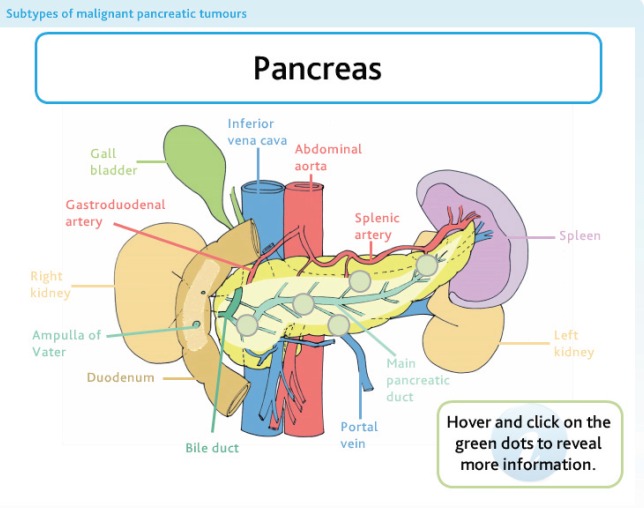
An interactive diagram used in the anatomy module.

**Figure 4.**
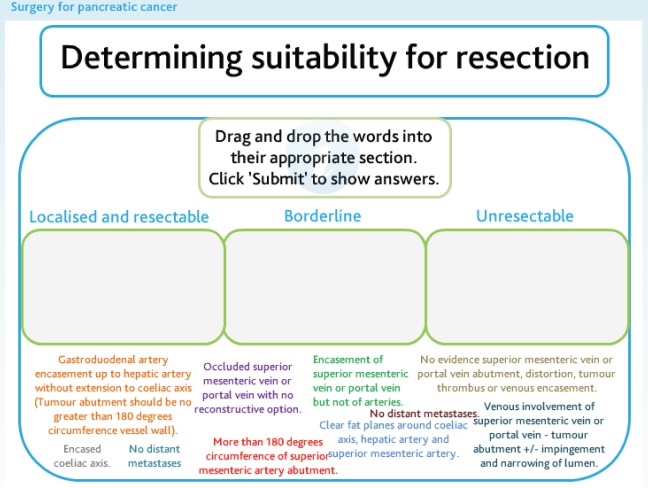
An interactive game used in the Surgical Management module.

**Figure 5.**
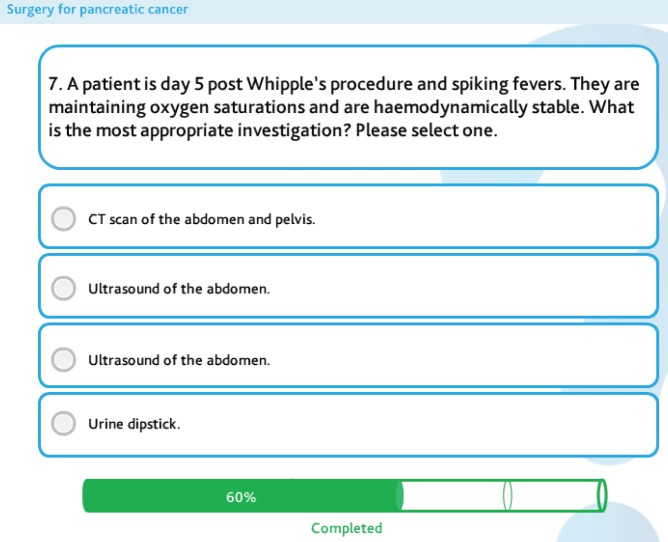
An example of a multiple-choice question in the pre-and post-module assessment.

Five modules were created, with each module covering a separate but linked aspect of pancreatic cancer (Figure 2). All modules begin with a multiple-choice assessment to gauge prior knowledge. The learner is then guided through the learning content, with each module using a variety of learning techniques, including video of operations and expert opinion, interactive diagrams (Figure 3), case examples, and games (Figure 4). All of which are aimed at ensuring that any type of learner can actively engage in the process. Each module concludes with a further assessment, which the learner is required to pass prior to progressing (Figure 5).

The modules will be hosted with *e*cancer’s online learning platform. To ensure that the knowledge gained by completing the modules has been retained, the platform communicates with the learner at one and three months after completion of the module, with additional educational support offered.

The initial aim of the project was achieved by developing five innovative learning modules. The project has also included additional benefits to both the collaborator and the trainee.

For the collaborator, the project has been presented internationally at the Royal Society of Medicine Conference on Innovation Driving Patient Care: Simulation, Robotics and Telemedicine, the Association of Surgeons of Great Britain and Ireland and the Association for the Study of Medical Education. The project has been presented to the Joint Committee on Surgical Training e-learning and simulation working group to discuss how this project may be developed further for the surgical trainees nationally.

The trainees have been able to develop medical education skills including taking part in formal training courses. The trainees also reported a number of transferable skills that they have developed, including teamwork, leadership, management, and an understanding of how to complete projects on time.

## Discussion

Oncology is a challenging subject matter not only for any learner, but also for the educator. Our *e*POSSOM collaborative has been created to develop educational material that combines the most up-to-date evidence and debate being discussed by world experts and research groups, with a combination of learning techniques that a modern online learning utilises.

Until we receive feedback from our learners to the initial modules, we obviously cannot claim them to be a success, but the collaboration is confident that we are providing unique learning experience to each learner.

The concept of e-learning is not new, and there are a number of other learning resources available online. The Royal College of Surgeons offers core trainees an e-learning package that it developed in collaboration with e-leaning for Healthcare [4]. While a number of medical education providers offer online learning resources for their students [5], these modules differ from the *e*POSSOM collaborative as they are not designed by trainees, are not evidence based or open access, and do not aim at the higher surgical trainee level. Web-based learning is also provided by other online cancer-specific sites [6]; however, these are not the modular learning program that have been developed by. They do not offer the extent of knowledge that our program does, or even coincide with a learning curriculum that ours does. More recently, organisations have begun to offer an interactive web-based expert seminar, or webinar, service to their members [7, 8]. These services may not be free or open access and only cover specific areas at specific times. They are also limited to the learning styles that can be applied. The *e*POSSOM approach, however, is able to cover a wider area of subject matter, be freely accessible at anytime that suits the trainee, and uses a variety of learning styles.

These modules aim to provide a unique learning experience in a subject area not tackled before in a similar way. Their unique selling point is the backing up of the content with evidence that has been resourced and condensed in a user-friendly manner. By referencing the content, the learner has the opportunity for reading further if they are specifically interested. Furthermore, the content is peer reviewed and so can be considered reliable in both its content and delivery methods.

The collaboration recognises that in order to maintain true to its aims, the modules must be regularly reviewed and updated, thus ensuring the modules remain relevant and useful to the learner. This is an area in which the collaboration has started implementing plans to ensure it is successful, and we believe that with time we are able to adapt our modules in a more flexible way than any printed text.

Collaborative work within the surgical fraternity is increasing in popularity. In the UK, there are networks of surgical trainees developing high-quality collaborative research projects [9, 10]. We believe this is the first educational collaborative publishing material in UK. Collaboration teaches trainees valuable skills in leadership and teamwork that are essential attributes to the modern consultant surgeon in the National Health Service (NHS).

The additional skills the *e*POSSOM collaborative team has acquired through experience should not be underestimated. For the trainees, it is insightful to see how an organisation like *e*cancer works and the resources they have available. The *e*cancer education team has the benefit of working directly with their end users and so can gain insight into what trainees want from their resources. The *e*POSSOM project is an example of collaboration that works for all.

Leadership is one of the hardest skills to teach and learn, and hands on experience is invaluable [11]. This project has allowed the development of these skills for all concerned and especially for those in the project lead roles. This is especially important for trainees who need to gain leadership and management experience, and training prior to gaining the certificate on completion of training.

## Conclusion

E-learning is not a new concept, nor is evidence-based medicine or collaboration. However, this project is novel and unique one in its combination of all three. *e*POSSOM creates innovative e-learning material for audiences across the world, allowing each learner access to complex evidence-based medicine, wherever their learning environment allows them.

The *e*POSSOM project also creates an innovative learning environment of essential other transferable skills for its collaborators. Speaking on behalf of the *e*POSSOM collaborative, it is a project we hope all are able to enjoy and learn from, just like we have.

## Resources

Introduction and overview of pancreatic cancer:

http://ecancer.org/education/module/150-introduction-and-overview-of-pancreatic-cancer/player.php

Investigations including screening controversies:

http://ecancer.org/education/module/151-investigations-including-screening-controversies.php

Oncology issues and palliative care:

http://ecancer.org/education/module/152-oncology-issues-and-palliative-care.php

Subtypes of malignant pancreatic tumours:

http://ecancer.org/education/module/153-subtypes-of-malignant-pancreatic-tumours.php

Surgery for pancreatic cancer:

http://ecancer.org/education/module/154-surgery-for-pancreatic-cancer.php

## Conflicts of interest

The authors declare that they have no conflicts of interest.

## Figures and Tables

**Table 1.** Members of the original *e*caner and Severn Postgraduate Deanery Collaborative.

**Professor Gordon McVie**	Chairman of *e*cancer, visiting Professor at King’s College London
**Danny Burke**	*e*cancer educational team
**Miss Katrina Butcher**	Specialist Trainee General Surgery
**Professor Richard Canter**	Head of School, Severn Deanery
**Mr Rob Longman**	Chair of Skills and Courses Severn Deanery, Consultant Colorectal and General Surgeon
